# Clinical Management of Movement Disorders

**DOI:** 10.3390/jcm13010043

**Published:** 2023-12-21

**Authors:** Lazzaro di Biase

**Affiliations:** Neurology Unit, Campus Bio-Medico University Hospital Foundation, Via Álvaro del Portillo 200, 00128 Rome, Italy; l.dibiase@policlinicocampus.it

## 1. Introduction

Movement disorders include a wide and heterogeneous variety of signs and syndromes, which are classified as hyperkinetic [1] and hypokinetic disorders [2]. Diagnoses of both hyperkinetic and hypokinetic movement disorders are still based on clinical evaluation [1,3]. Indeed, hyperkinetic movement disorders should be classified according to their time features (rhythmicity, speed, and duration of the muscular contraction), space characteristics (body distribution, muscular pattern, and amplitude of the movement), and body state (action/rest, eventual suppressibility, and wakefulness) [4]. Conversely, bradykinesia, rest tremors, and rigidity represent the cardinal motor symptoms for the diagnosis of the parkinsonian syndrome [3]. However, especially for Parkinson’s disease (PD), diagnostic accuracy is notably influenced by a significant error rate [5], which is related to the lack of objective biomarkers for the in vivo diagnosis of PD. A quantitative and objective analysis of motor performances appears to be a necessary approach [6,7,8]. For this purpose, the integration of Artificial Intelligence (AI) to smart devices has shown potential for improving diagnostic accuracy, facilitating both in-clinic and at-home assessments of motor performances of movement disorders [9,10]. Pertaining to diagnoses, smart devices have demonstrated the possibility of identifying a whole variety of motor symptoms, such as bradykinesia [11,12], rigidity [13,14], tremors [15,16,17,18], gait, balance and posture [12,19,20,21], and motor complications [22], even at home [23,24]. Nevertheless, the therapeutic side can also be frustrating due to a lack of information related to the whole-day motor state fluctuation, since the clinical visit can last from 30 min to 1 h and therefore can only evaluate a small piece of the day. However, a significant effort is still required to produce solid normative data for a clinical validation on a large scale.

In this Special Issue, we embark on a journey through the multifaceted world of movement disorders, capturing the various contributions of studies that focus on improving both the diagnostic and the therapeutic aspects of movement disorders. This Special Issue contains seven articles and four reviews, which are briefly discussed in the next paragraph.

## 2. Overview of Published Articles

Clinical neurophysiology and genetics could help in the diagnostic process of one of the most studied hyperkinetic disorders, namely dystonia (Contributor 1). On this topic, Rogić Vidaković et al. (Contributor 2) propose a narrative review and case reports on focal laryngeal dystonia, highlighting innovative treatment approaches such as transcranial magnetic stimulation (TMS). Pinero-Pinto et al. (Contributor 3) present a narrative review, and discuss the interplay between motor skills and visual deficits in developmental coordination disorder, broadening our understanding of this complex condition. Regarding hypokinetic movement disorders, Heimrich et al. (Contributor 4) propose an innovative network analysis, which delves into the often-overlooked non-motor symptoms in Parkinson’s disease (PD) and their profound impact on patients’ quality of life. Adding a technological dimension to the diagnosis of Parkinson’s disease, di Biase et al. (Contributor 5) utilize a high-density EEG to explore brain connectivity in these patients, offering a novel diagnostic perspective. In light of objective symptom evaluations, Vilas-Boas et al. (Contributor 6) provide a fascinating case series on gait analysis using an optical system and force platforms in hereditary amyloidosis patients, a study that underscores the importance of comprehensive and quantitative symptom assessment. During recent decades, different advanced therapeutic techniques have been developed in order to also face motor problems, not only in terms of hyperkinetic movement disorders, but also of hypokinetic disorders like PD [25,26,27,28,29,30,31]. A well-established and innovative treatment for movement disorders is Deep Brain Stimulation (DBS), which has been found to be a successful application for the treatment of both various hyperkinetic movement disorders [32] and PD [33]. In terms of the treatment aspect of movement disorders, Hefter et al. (Contributor 7), shed light on the differential recovery timelines of brain and liver dysfunctions in Wilson’s Disease, a well-known cause of secondary dystonia, highlighting the complexities of treatment responses. On the same topic, Woimant et al. (Contributor 8) examine Trientine salts in the treatment of Wilson’s Disease, offering hope for management strategies. In a similar vein to addressing treatment challenges, González-Herrero et al. (Contributor 9) retrospectively study and critically evaluate the treatment options for dystonic tremors of the upper limbs, a condition that often puzzles clinicians. In a shift towards treatment innovations, Szczakowska et al. (Contributor 10) review the role of Deep Brain Stimulation (DBS) in managing tardive dyskinesia, a challenging and often distressing condition. Finally, Kim et al. (Contributor 11) provide valuable insights into the treatment changes and prognoses of drug-induced parkinsonism, a pressing concern in clinical practice, especially in South Korea.

## 3. Future Directions

Together, these papers not only advance our understanding of movement disorders, but also open new avenues for research and clinical practice. Different strategies have been proposed by the authors, ranging from neurophysiological to machine learning approaches. However, new therapeutic insights for movement disorders will probably arise from the evidence of sensibility of the neurons to not only pharmacological stimulation, but also to electrical [34], magnetic [35], or ultrasound stimulations [36]. Beyond the objective analyses, the possibility of real-time monitoring of a patient affected by a movement disorder (especially PD) throughout the course of the whole day could also increase the reliability of clinical assistance. Knowledge of the motor state variability of such a complex patient during the day could lead to tailored therapy, avoiding motor complications such as levodopa-induced dyskinesias or underdosed levodopa regimens [37,38]. Biochemical, neurophysiological, and wearable sensor data could be part of a multiparametric modular sensing system that regulates therapy according to the motor state [39,40].

## List of Contributors

1. di Biase, L.; Di Santo, A.; Caminiti, M.L.; Pecoraro, P.M.; Carbone, S.P.; Di Lazzaro, V. Dystonia diagnosis: clinical neurophysiology and genetics. *J. Clin. Med.* **2022**, *11*, 4184. https://doi.org/10.3390/jcm11144184.
2. Rogić Vidaković, M.; Gunjača, I.; Bukić, J.; Košta, V.; Šoda, J.; Konstantinović, I.; Bošković, B.; Bilić, I.; Režić Mužinić, N. The patho-neurophysiological basis and treatment of focal laryngeal dystonia: a narrative review and two case reports applying TMS over the laryngeal motor cortex. *J. Clin. Med.* **2022**, *11*, 3453. https://doi.org/10.3390/jcm11123453.
3. Pinero-Pinto, E.; Romero-Galisteo, R.P.; Sánchez-González, M.C.; Escobio-Prieto, I.; Luque-Moreno, C.; Palomo-Carrión, R. Motor Skills and Visual Deficits in Developmental Coordination Disorder: A Narrative Review. *J. Clin. Med.* **2022**, *11*, 7447. https://doi.org/10.3390/jcm11247447.
4. Heimrich, K.G.; Schönenberg, A.; Santos-García, D.; Mir, P.; COPPADIS Study Group; Prell, T. The Impact of Nonmotor Symptoms on Health-Related Quality of Life in Parkinson’s Disease: A Network Analysis Approach. *J. Clin. Med.* **2023**, *12*, 2573. https://doi.org/10.3390/jcm12072573.
5. di Biase, L.; Ricci, L.; Caminiti, M.L.; Pecoraro, P.M.; Carbone, S.P.; Di Lazzaro, V. Quantitative High Density EEG Brain Connectivity Evaluation in Parkinson’s Disease: The Phase Locking Value (PLV). *J. Clin. Med.* **2023**, *12*, 1450. https://doi.org/10.3390/jcm12041450
6. Vilas-Boas, M.D.C.; Fonseca, P.F.P.; Sousa, I.M.; Cardoso, M.N.; Cunha, J.P.S.; Coelho, T. Gait Characterization and Analysis of Hereditary Amyloidosis Associated with Transthyretin Patients: A Case Series. *J. Clin. Med.* **2022**, *11*, 3967. https://doi.org/10.3390/jcm11143967.
7. Hefter, H.; Kruschel, T.S.; Novak, M.; Rosenthal, D.; Luedde, T.; Meuth, S.G.; Albrecht, P.; Hartmann, C.J.; Samadzadeh, S. Differences in the time course of recovery from brain and liver dysfunction in conventional long-term treatment of Wilson disease. *J. Clin. Med.* **2023**, *12*, 4861.
8. Woimant, F.; Debray, D.; Morvan, E.; Obadia, M.A.; Poujois, A. Efficacy and Safety of Two Salts of Trientine in the Treatment of Wilson’s Disease. *J. Clin. Med.* **2022**, *11*, 3975.
9. González-Herrero, B.; Di Vico, I.A.; Pereira, E.; Edwards, M.; Morgante, F. Treatment of Dystonic Tremor of the Upper Limbs: A Single-Center Retrospective Study. *J. Clin. Med.* **2023**, *12*, 1427.
10. Szczakowska, A.; Gabryelska, A.; Gawlik-Kotelnicka, O.; Strzelecki, D. Deep brain stimulation in the treatment of tardive dyskinesia. *J. Clin. Med.* **2023**, *12*, 1868.
11. Kim, S.; Suh, H.S. Treatment Changes and Prognoses in Patients with Incident Drug-Induced Parkinsonism Using a Korean Nationwide Healthcare Claims Database. *J. Clin. Med.* **2023**, *12*, 2860.
